# The Role of Force Fields and Water Models in Protein Folding and Unfolding Dynamics

**DOI:** 10.1021/acs.jctc.3c01106

**Published:** 2024-02-19

**Authors:** Anna-Lena M. Fischer, Anna Tichy, Janik Kokot, Valentin J. Hoerschinger, Robert F. Wild, Jakob R. Riccabona, Johannes R. Loeffler, Franz Waibl, Patrick K. Quoika, Philipp Gschwandtner, Stefano Forli, Andrew B. Ward, Klaus R. Liedl, Martin Zacharias, Monica L. Fernández-Quintero

**Affiliations:** † Institute for General, Inorganic and Theoretical Chemistry, Center for Molecular Biosciences Innsbruck (CMBI), University of Innsbruck, A-6020 Innsbruck, Austria; ‡ Department of Chemistry and Applied Biosciences, ETH Zürich, Vladimir-Prelog-Weg 2, 8093 Zürich, Switzerland; § Center for Protein Assemblies (CPA), Physics Department, Chair of Theoretical Biophysics, Technical University of Munich, D-80333 Munich, Germany; ∥ Research Center HPC, University of Innsbruck, A-6020 Innsbruck, Austria; ⊥ Department of Integrative Structural and Computational Biology, Scripps Research Institute, La Jolla, California 92037, United States

## Abstract

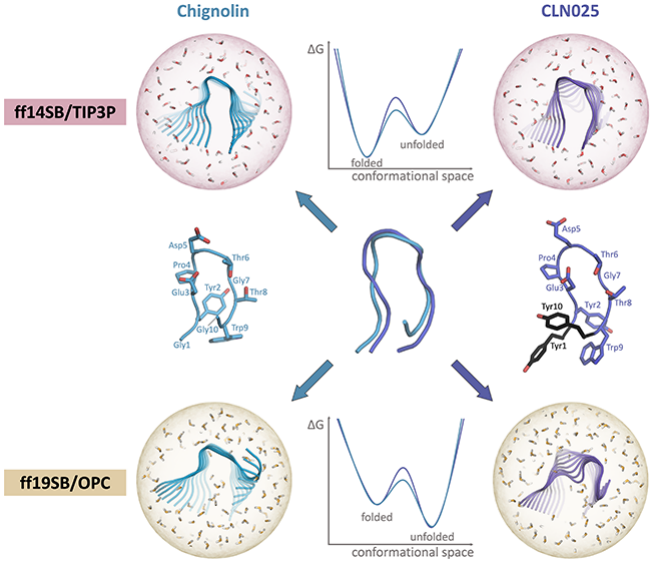

Protein folding is a fascinating, not fully understood phenomenon in biology. Molecular dynamics (MD) simulations are an invaluable tool to study conformational changes in atomistic detail, including folding and unfolding processes of proteins. However, the accuracy of the conformational ensembles derived from MD simulations inevitably relies on the quality of the underlying force field in combination with the respective water model. Here, we investigate protein folding, unfolding, and misfolding of fast-folding proteins by examining different force fields with their recommended water models, i.e., ff14SB with the TIP3P model and ff19SB with the OPC model. To this end, we generated long conventional MD simulations highlighting the perks and pitfalls of these setups. Using Markov state models, we defined kinetically independent conformational substates and emphasized their distinct characteristics, as well as their corresponding state probabilities. Surprisingly, we found substantial differences in thermodynamics and kinetics of protein folding, depending on the combination of the protein force field and water model, originating primarily from the different water models. These results emphasize the importance of carefully choosing the force field and the respective water model as they determine the accuracy of the observed dynamics of folding events. Thus, the findings support the hypothesis that the water model is at least equally important as the force field and hence needs to be considered in future studies investigating protein dynamics and folding in all areas of biophysics.

## Introduction

Proteins are essential macromolecules throughout the kingdom of life. Due to their high variability in sequence and structure they can fold into a panoply of different structures and fulfill various different roles and functions, e.g., in metabolism,^1,2^ signal transduction,^3,4^ immunity,^5,6^ muscle contraction,^7,8^ or drug delivery.^9,10^ Protein-based therapeutics, also known as biologics, are an increasingly important class of drugs, which has been highlighted in various previous studies.^11−14^ Hence, understanding protein folding, unfolding, and misfolding is an important ongoing field of research.^15−17^ It is well-known that most proteins can adopt a distinct fold, which is evolutionarily well conserved, even more than their sequence.^18^ However, it still remains elusive why a specific sequence adopts a certain fold.^16,19^ The accurate prediction of protein folding and unfolding dynamics is crucial for numerous fields of research, including drug design,^20,21^ enzyme engineering,^22^ and understanding the molecular basis of diseases.^23,24^ After all, certain conformational rearrangements, such as misfolds, are expected to be involved in numerous diseases, like Alzheimer’s disease, Huntington disease, Parkinson’s disease, or diabetes type 2.^25−28^ Additionally, refolding events resulting from point mutations have been shown to elucidate changes in specificity or even polyreactivity, providing new applications in drug design and engineering.^29−32^

In recent years, fast-folding proteins have been in the focus of various studies.^33−35^ These proteins provide an excellent basis for studying the principles of protein folding, since they allow us to capture folding events in the micro- to millisecond time scale. Furthermore, they exhibit simplified folding dynamics and therefore offer insights into the fundamental interactions and energy landscapes that drive the folding process.

**Figure 1:**
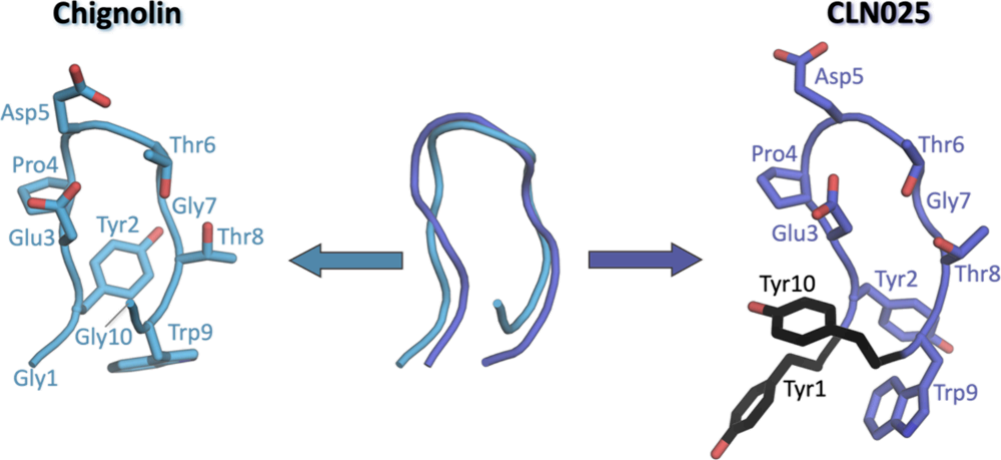
Schematic representation of the investigated systems: on the left is Chignolin (PDB 1UAO),^37^ and on the right is CLN025 (PDB 5AWL)^39^ depicted. The mutated residues in CLN025 are highlighted in black (Tyr1 and Tyr10). In the middle, an overlay of both experimentally determined structures used as starting structures for our simulations is shown.

In this study, we focus on the fast-folding protein Chignolin^36^ and its variant CLN025^37^ (Figure 1). These short proteins both consist of ten amino acids and adopt a β-hairpin structure as their native fold. They only differ in their terminal residues, namely, the glycines in Chignolin are replaced with two tyrosines in CLN025, which have been reported to increase the stability of the folded state.^37,38^

Besides experimental techniques such as NMR, fluorescence measurements, cDNA display proteolysis, and many more,^40−43^ molecular dynamics (MD) simulations represent an invaluable addition to characterize and study folding processes at atomistic level. Unfortunately, a simulation is only as good as the input parameters used. This fact drives the ongoing development (and improvement) of force fields, water models, and other parameters. The two AMBER^44^ force fields ff14SB^45^ and ff19SB^46^ have demonstrated good agreement to experimental data in protein studies and thus are among many other popular force fields extensively applied in MD simulations.^47^ While the ff14SB force field includes small empirical adjustments based on NMR data via parametrization with TIP3P,^48^ ff19SB is solely physics-based and therefore does not depend on a distinct water model. However, the developers recommend the usage of OPC^49^ (2014) as it reproduces water properties more accurately than older models like TIP3P (1981). Although the AMBER^44^ force fields are widely used, other options should also be considered. Especially CHARMM,^50,51^ GROMOS,^52^ and OPLS^53^ are noteworthy alternatives, as reflected in their increasing utilization. Beyond conventional force fields, polarizable force fields may potentially improve simulation accuracies, however, at significantly increased computational cost.^35,54^ Here, we only consider non-polarizable force fields.

The water model has a large impact on the simulated protein properties. The TIP3P (transferable intermolecular potential 3-point) water model, developed by Jorgensen et al., approximates water behavior using a three-site representation.^48^ It has been widely used in protein simulations due to its simplicity and computational efficiency compared to other 3-point (SPC,^55,56^ SPC-E,^57^ OPC3,^58^ etc.) or 4-point water models (TIP4P,^59^ TIP4Pew,^60^ etc.). In recent years, the four-site OPC (optimal 4-point 3-charge rigid water model) model has gained attention among others as an alternative solvent model because it describes bulk water more accurately than less modern water models. It was parametrized based on charge optimization instead of constrained structural features during parametrization.^49^ OPC has demonstrated better performance in reproducing experimental thermodynamic properties and water–water hydrogen bonding interactions compared to TIP3P.^49^

Here, we contribute to the ongoing exploration of protein folding dynamics by providing a detailed comparative analysis of ff14SB/TIP3P and ff19SB/OPC by means of fast-folding proteins, i.e., Chignolin and its mutant CLN025. We highlight strengths and limitations of both force fields in combination with their corresponding water models and compare the folding kinetics and thermodynamics with respect to the existing experimental data.

## Methods

### Simulation Setup

We chose Chignolin (PDB 1UAO)^37^ and its mutant CLN025 (PDB 5AWL)^39^ to compare the influence of the force fields (ff14SB and ff19SB) and the water models (TIP3P and OPC, respectively) on folding thermodynamics and kinetics. The structures were prepared in MOE (molecular operating environment)^61^ applying the Protonate 3D tool (all amino acids were in their standard protonation state at pH 7).^62^ We placed the fast-folding proteins into cubic water boxes (of TIP3P^59^ or OPC^49^ water molecules, respectively) with a minimum wall distance to the protein of 20 Å.^63,64^ To this end, we used the tleap tool from the AmberTools20 package.^65^ Parameters for the simulations were derived either from the AMBER force field 14SB^46^ (for TIP3P water) or 19SB^45^ (for OPC water). To neutralize the charges, we used uniform background charges^65−67^ and each system was carefully equilibrated using a multistep equilibration protocol.^68,69^

With pmemd.cuda, MD simulations in an NpT ensemble were performed.^70^ Long range electrostatic interactions were calculated using the particle-mesh Ewald method. Bonds involving hydrogen atoms were restrained according to the SHAKE algorithm, enabling a 2.0 fs time step.^71^ The atmospheric pressure (1 bar) of the system was kept constant by weak coupling to an external bath using the Berendsen algorithm.^72^ The Langevin thermostat was used to maintain the temperature during simulations at 300 K.^73^ For each system, two distinct simulations, each of 6 μs, were performed. The long trajectories were clustered in 2D root mean squared deviation (2D-RMSD) according to a hierarchical agglomerative approach implemented in cpptraj^66^ (σ = 1.8 Å). We started new simulations of every cluster representative (100 ns) to enhance the coverage of the conformational surface (Table 1).

**Table 1:** Detailed Description of the Simulated Systems and Aggregated Simulation Times

system	force field/water model	total aggregated simulation time
Chignolin	ff14SB/TIP3P	61.5 μs
Chignolin	ff19SB/OPC	110.9 μs
CLN025	ff14SB/TIP3P	35.7 μs
CLN025	ff19SB/OPC	82.2 μs

### Simulation Analysis

With the obtained trajectories we performed a time-lagged independent component analysis (tICA) using the Python library PyEMMA 2 employing a lag time of 5 ns.^74^ Thermodynamics and kinetics were recovered by Markov state models (MSMs) using PyEMMA 2.^75^ Herein, a *k*-means clustering algorithm^76^ was used to define microstates and a PCCA+ clustering algorithm^77^ was used for coarse-graining the microstates into macrostates. In general, Markov state models produce a coarse-grained representation of the kinetically distinct conformational states and enable the reconstruction of the free energy surface. The Chapman–Kolmogorov test^78,79^ evaluates the sampling efficiency and the reliability of the model by employing the variational approach for Markov processes and by accounting for the fraction of states used.^80^ To build the Markov state model, we used the distances between all heavy atoms, defined 100 microstates using the *k*-means clustering algorithm, and applied a lag time of 5 ns.

To calculate intramolecular contacts of the investigated proteins, we used the GetContacts software.^81^ The formation and duration of the respective contacts during a simulation can be visualized with so-called flareplots. In these plots, interacting residues are connected via a line that is colored according to the frequency of the interaction. To visualize the contacts, we used an in-house Python script, which is available on GitHub (https://github.com/liedllab/GetContacts_analysis) and the Python library MNE-connectivity (https://github.com/mne-tools/mne-connectivity). We focused our analysis on hydrogen bond interactions and contacts involving aromatic amino acids (π-stacking and T-stacking). Furthermore, secondary structures, RMSDs (of the Cα atoms), and native contacts were calculated using the AMBER implementation cpptraj.^66^ As reference, both for the RMSD and the native contacts, the experimental structures (Chignolin: PDB 1UAO,^37^ CLN025: PDB 5AWL(39)) were used. The secondary structure assignment has been performed according to DSSP,^82^ and error estimates were evaluated with block averaging.

### Grid Inhomogeneous Solvation Theory

Grid inhomogeneous solvation theory (GIST) was used to estimate localized thermodynamic properties of solvation.^83−86^ GIST is an MD-based approach originating from inhomogeneous solvation theory (IST), which considers the solvation process in terms of localized effects on the solvent around the solute compared to the bulk solvent.^87,88^ GIST replaces IST’s spatial integrals describing the solvation thermodynamics through discrete sums over a three-dimensional grid, simplifying the underlying calculations. In this study, we apply GIST to calculate the free energy of solvation for five random sample structures of each MSM macrostate to estimate the ensemble properties. Simulations (100 ns) were run with a similar simulation setup as described above; however, all considered protein structures were solvated into a solvent box with a side length of 75 Å. During the simulation, the protein heavy atoms were kept rigid by applying harmonic position restraints of 1000 kcal mol^–1^ Å^–2^. A GPU-accelerated version of the GIST algorithm^89,90^ was used to calculate thermodynamic properties of the last 80 ns of each trajectory on a grid with a size of 75 × 75 × 75 Å and a voxel spacing of 0.5 Å at a temperature of 300 K. Reference densities of 0.0329 and 0.0333 molecules Å^–3^ were used for the TIP3P and the OPC water models, respectively, according to their bulk density. The free energy of solvation for voxel *k* is calculated as follows, including a recently suggested correction factor of 0.4 Δ*S* to account for the missing higher-order entropy terms:^90,91^
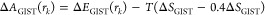
1Here, the GIST entropy Δ*S*_GIST_ (*r*_*k*_) of a single voxel is derived from orientational and translational mobility of the water molecules within this voxel during the simulation. To this end, the distribution of orientations and populations of water molecules within the respective voxel is evaluated over a short simulation. Furthermore, the GIST enthalpy Δ*E*_GIST_ (*r*_*k*_) of the water molecules within a single voxel quantifies the average interaction of these waters to all other voxels and the solute. For each single peptide structure, thermodynamic properties were calculated by integrating over all GIST voxels within 9 Å of the structures according to Kraml et al.^92^ To get an ensemble value for the macrostate free energy of solvation, the arithmetic average for each state was calculated from the respective single values.^14^ In general, the entropy is derived from orientational and translational mobility of the water molecules, while the enthalpy quantifies the interaction between the water molecules in the solvation shell or the bulk solvent, respectively. Error estimates for the sampling errors were again evaluated with block averaging of the restrained GIST simulations.

## Results

We elucidate the influence of distinct force fields (ff14SB and ff19SB) and water models (TIP3P and OPC) on the folding characteristics of Chignolin and its mutant CLN025. We analyzed both kinetic and thermodynamic properties in our simulations. To ensure enough conformational transitions between folding and unfolding events, we aggregated in total 290 μs of sampling time (Table 1).

### Characterization of Conformational States

**Figure 2:**
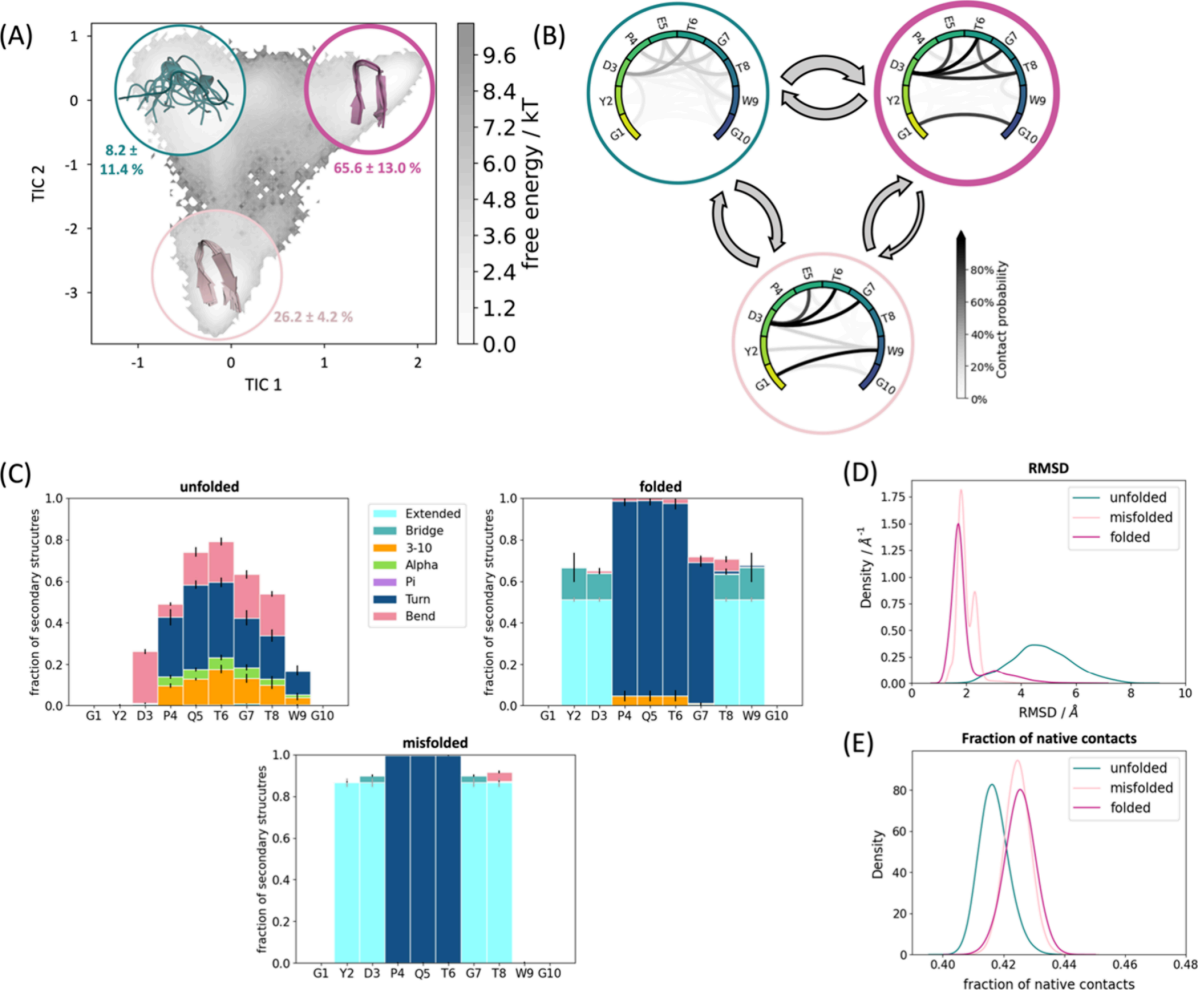
Detailed analysis of Chignolin simulated with ff14SB/TIP3P. (A) The population of the macrostates projected onto the free energy landscape. Green is the unfolded state, purple the natively folded, and light pink the misfolded state. The transition times and the MSM validations are shown in the SI. (B) The intramolecular interaction patterns of these respective states. The darker the interaction, the higher the occurrence in the simulation. The arrows between the states represent the transition times (for further details, see the SI). The larger the arrow, the faster the transition time. In general, the transition times all range in the 100 ns area. (C) The secondary structures for each state displayed determined according to DSSP.^82^ (D) The RMSDs and (E) the fractions of native contacts.

**Figure 3:**
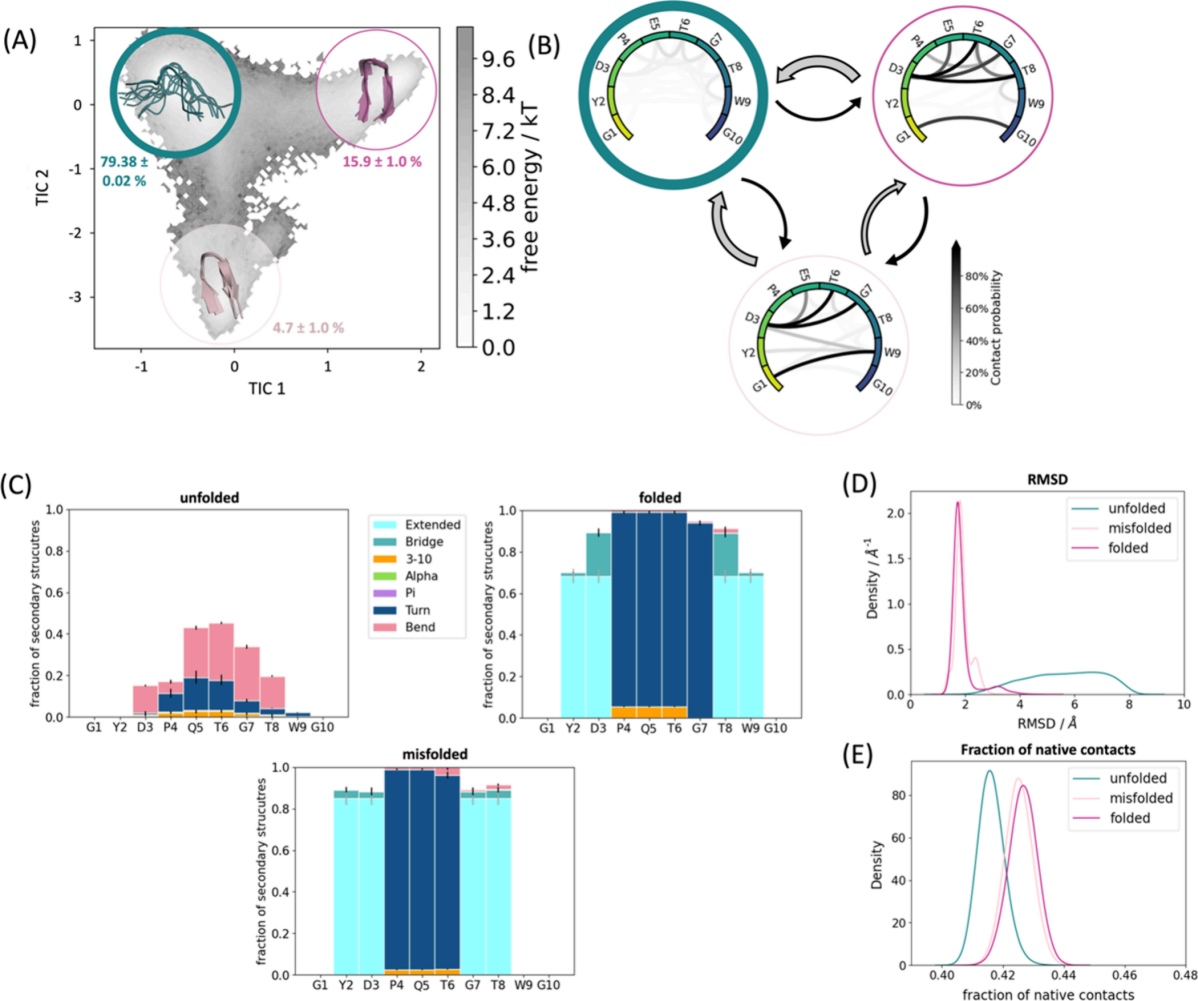
Detailed analysis of Chignolin simulated with ff19SB/OPC. (A) The population of the macrostates projected onto the free energy landscape. Green is the unfolded state, purple the natively folded, and light pink the misfolded state. The transition times and the MSM validations are shown in the SI. (B) The intramolecular interaction patterns of the respective states. The darker the interaction, the higher the occurrence in the simulation. The arrows between the states represent the transition times (for further details, see the SI). The larger the arrow, the faster the transition time. In general, the transition times all range in the 100 ns (gray arrows) to low microsecond (black arrows) area. (C) The secondary structures for each state displayed determined according to DSSP.^82^ (D) The RMSDs and (E) the fractions of native contacts.

**Figure 4:**
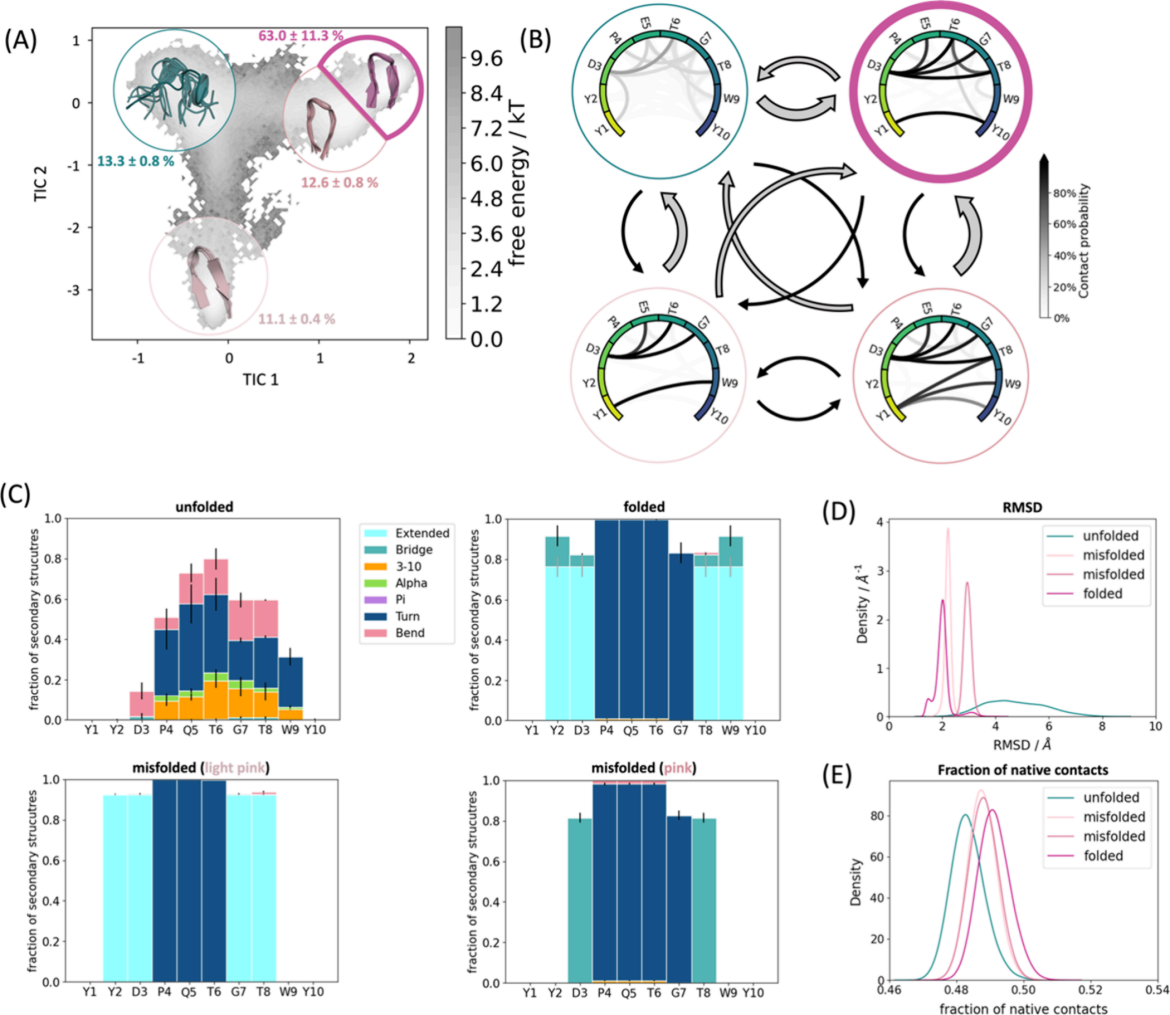
Detailed analysis of CLN025 simulated with ff14SB/TIP3P. (A) The population of the macrostates projected onto the free energy landscape. Green is the unfolded state, purple is the natively folded, and pink and light pink are the misfolded states. The transition times and the MSM validations are shown in the SI. (B) The intramolecular interaction patterns of the respective states. The darker the interaction, the higher the occurrence in the simulation. The arrows between the states represent the transition times (for further details, see the SI). The larger the arrow, the faster the transition time. In general, the transition times all range in the 100 ns (gray arrows) to low microsecond (black arrows) area. (C) The secondary structures for each state displayed determined according to DSSP.^82^ (D) The RMSDs and (E) the fractions of native contacts.

**Figure 5:**
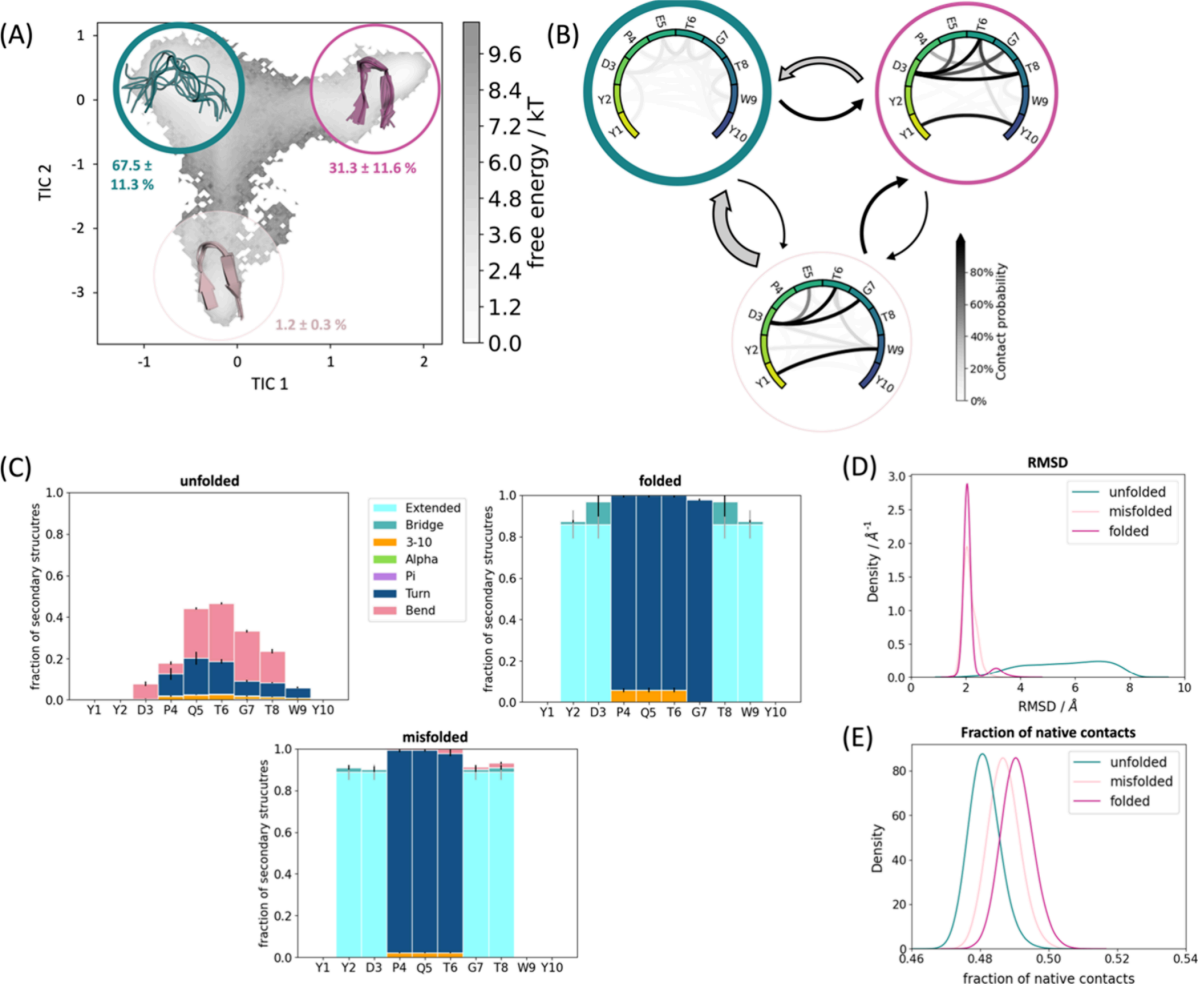
Detailed analysis of CLN025 simulated with ff19SB/OPC. (A) The population of the macrostates projected onto the free energy landscape. Green is the unfolded state, purple the natively folded, and light pink the misfolded state. The transition times and the MSM validations are shown in the SI. (B) The interaction patterns of the respective states. The darker the interaction, the higher the occurrence in the simulation. The arrows between states represent the transition times. The larger the arrow, the faster the transition time (for further details, see the SI). In general, the transition times all range in the 100 ns (gray arrows) to low microsecond (black arrows) area. (C) The secondary structures for each state displayed determined according to DSSP.^82^ (D) The RMSDs and (E) the fractions of native contacts.

Figures 2, 3, 4, and 5 (A) show the free energy surface in terms of the first two TICA coordinates for Chignolin and CLN025. In these free energy landscapes, we show the Markov state models of the folding dynamics, discretizing the free energy surface in three to four kinetically separated conformational states on the free energy landscape. In both Chignolin (Figures 2 and 3) and CLN025 (Figures 4 and 5), independent of the force fields and water models, we found a clear separation of folded and unfolded states. The unfolded state representatives are always shown in green, while the folded state conformations are depicted in purple (Figures 2–5). The latter can be further split up in accordance with available crystal/NMR structures into native folded (purple) and misfolded states (pink and light pink). In Figures 2–5 (parts D and E) we find significant differences in the RMSDs and fraction of native contacts (abbreviated with *q*) underlining the distinct conformations of the defined states. While the native folded state shows the lowest RMSD (ca. 2 Å) and the highest *q* compared to the experimental data, the unfolded state shows the exact opposite trend bearing high RMSD and low *q*. However, the difference between the natively folded and the misfolded states are less pronounced (purple, pink, and light pink states).

Remarkably, the secondary structures of the different folded states (Figures 2–5 (C)) are clearly different: the misfolded state occurrent in all simulations (light pink) highlights a shifted hairpin conformation (extended β-sheet in cyan) compared to the native folded structure, where G7–D3 and T8–Y2 form the β-sheet instead of T8–D3 and W9–Y2. The second misfolded state, which could only be separated in the CLN025 simulations with ff14SB/TIP3P (Figure 4 (C), pink), is dominated by the turn structure (dark blue) of the central residues (4–7) but is missing the extended β-sheet conformation (only a bridge is occurring, colored medium blue). The unfolded state does not adopt any definite secondary structure but shows low occurrence of all different patterns.

A detailed look at the interaction patterns of the states reveals a more complex network in the native fold (Figures 2–5 (B, purple)). Hydrogen bond formation hints at a higher number of interaction partners in the β-sheet region compared to the misfolded states, which also stabilizes this secondary structure. Nevertheless, the misfolded states also have a noteworthy number of interactions that are more conserved between specific binding partners. Of significant importance for differentiation of the native and misfolded states seem to be the hydrogen bond interaction counterparts of residue 1 and residue 3 evident in Figures 2–5 (B, light pink). As for the native state, these residues form interactions that characterize the symmetry of the structure (G1/Y1–G10/Y10 and D3–T8). The misfolded states do not show this symmetry by forming shifted hydrogen bonds from G1/Y1 to W9 instead of G10/Y10. Similarly, D3–G7 is present instead of D3–T8. Furthermore, the additional misfolded state (Figure 4B, pink) shows higher variability in stabilizing interaction partners, especially highlighting increased side chain hydrogen bonds. This characterizes the shifted conformation of this state, where the steric character of the tyrosines causes distorted ends of the protein chain (also visible in the structural representation in Figure 4A).

### Effect of Force Field and Water Model on Protein Structure

Even though the same conformational space (Figure S1) is covered by all simulations, the populations of the different conformations and transition kinetics vary significantly. While the simulations with ff14SB/TIP3P show the native folded to be the highest populated state (Figures 2 and 4 (A)), the ff19SB/OPC simulations reveal a population shift toward the unfolded state, which becomes the highest populated (Figures 3 and 5 (A)). In addition, the transition times are affected.

Furthermore, ff14SB/TIP3P shows a higher bias toward helix formation (around 7% compared to 3% for ff19SB/OPC in Chignolin as well as in CLN025 averaged over all residues during the whole simulation; Figures 2–5 (C)). Interestingly, this secondary structure motif is not separable from the unfolded Markov state. Also, the interaction occurrence demonstrates a higher number of hydrogen bonds on average in the more stabilized ff14SB/TIP3P simulations (14 hydrogen bonds on average compared to four in ff19SB/OPC simulations; see Figure S3).

### Structural Comparison of Chignolin and CLN025

The high similarity in sequence, in addition to the short length of Chignolin and CLN025, renders the observed similarity in conformational space unsurprising. However, the additional stabilization due to the terminal tyrosines in CLN025 compared to glycines in Chignolin is prevalent in our analyses. We find an increase in the number of folded conformations for CLN025 independent of the force field/water model combinations. Furthermore, the misfolded states simultaneously become destabilized in the CLN025 simulations, ensuring a higher prevalence of the native folded structure. A detailed look into the interaction patterns of the residues forming the folded states (Figures 4 and 5 (B)) reveals the crucial impact of the terminal tyrosines in stabilizing the folded conformation. Owing to their aromatic character and π-stacking interactions between these two amino acids, Y2 and W9 form a hydrophobic patch sustaining the β-hairpin. This distinct characteristic is less pronounced in Chignolin since it is missing the terminal aromatic residues.

### Solvation Thermodynamics

**Figure 6:**
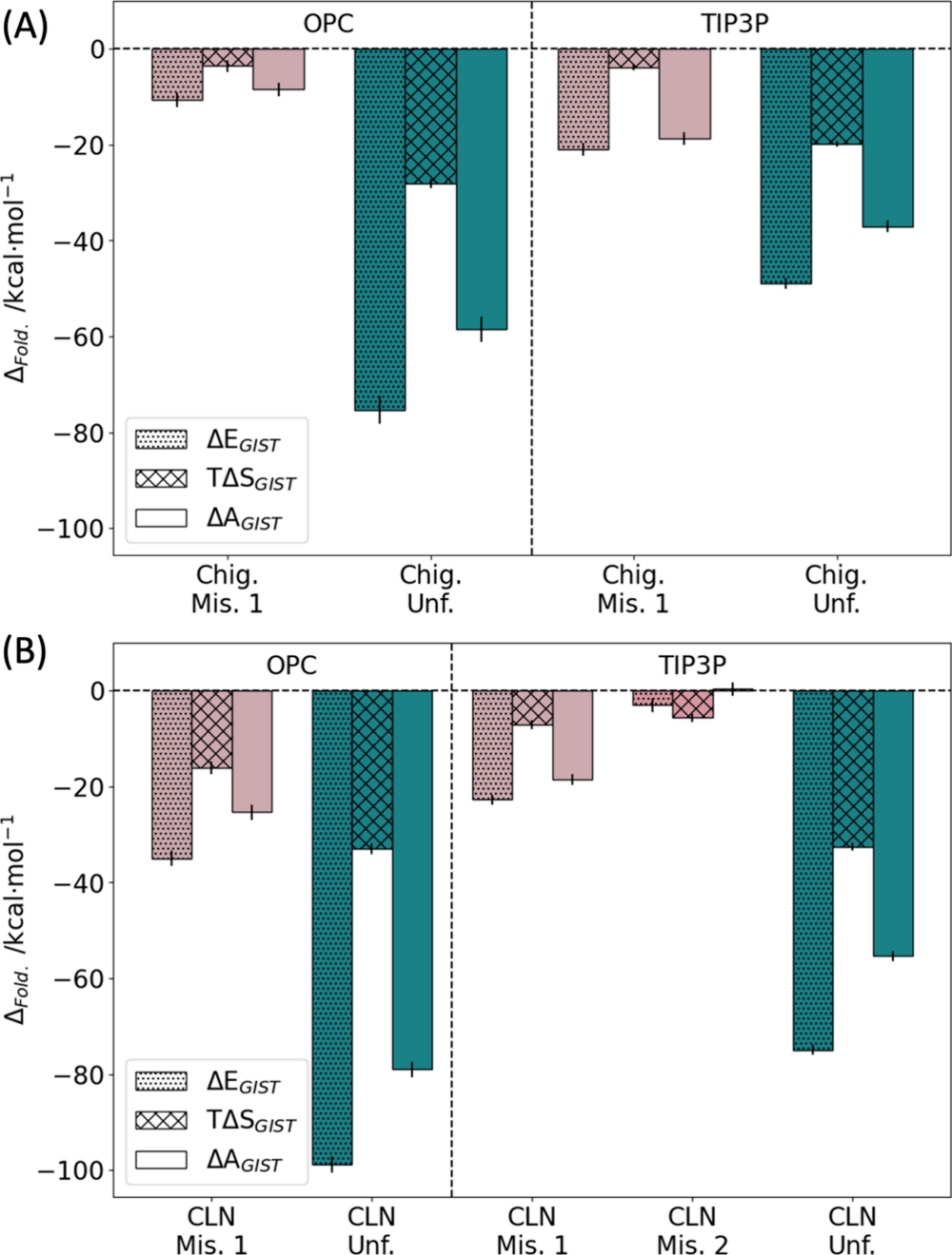
Differences in GIST properties of misfolded (Mis. 1 and Mis. 2, colored pink and light pink) and unfolded (Unf., colored green) states to folded states for (A) Chignolin (Chig.) and (B) mutant CLN025 (CLN). Left side: for the OPC water model. Right side: for the TIP3P water model. Displayed are the differences of the free energy of solvation (Δ*A*_GIST_, no pattern) and its components, the mean solvation enthalpy (Δ*E*_GIST_, dotted), and the solvation entropy (*T*Δ*S*_GIST_, crossed) calculated from eq 1. The error bars give the sampling error within the GIST simulations, as assessed by block averaging for each trajectory and error propagation to the combined ensemble properties.

The difference of the mean thermodynamic properties of the mis- and unfolded states to the folded state shown in Figure 6 highlights that for all combinations of force fields, the water environment favors the unfolded state the most, followed by the misfolded states. The folded state provides the least favorable environment for water. This is expected, as there is always a balance to be struck between peptide–peptide and peptide–water interactions. An unfavorable change in solvation free energy between two states is often counteracted by even more favorable intramolecular interactions, e.g., through the formation of stronger hydrogen bonds than would be possible with water. Indeed, this counterbalance is at the core of protein secondary structure and without it, no fold would be stable in solution.^93^ Comparing the variations to the folded states of CLN025 and Chignolin, the mutant shows larger differences in terms of solvation free energy. This results in stronger stabilization of the unfolded and misfolded states by the solvent. Most likely, this arises from the introduction of the two hydroxy groups on the mutants’ additional tyrosines. Remarkably, when investigating the difference between the two water models, the OPC is found to favor the unfolded and to a lesser degree the misfolded state more than TIP3P. Furthermore, the protein–water network (data not shown) reveals more stabilized interactions in the ff19SB/OPC simulations. In sum, these calculations indicate that OPC overstabilizes the unfolded state in comparison to TIP3P, in line with the observations from the MSMs. We find conclusive results in the thermodynamic contributions to the solvation free energy: The difference between the water models seems to arise from the enthalpy, where the variation is noticeably larger than that for the entropies. In terms of the dynamics, the OPC water therefore seems to behave similarly to TIP3P water but results in stronger interactions. We find that the entropy of solvation is systematically smaller in the OPC, however, the differences between the two water models are small. In contrast, the enthalpies of solvation are significantly different. From this observation, we reason that, in comparison to bulk solvent–solvent interactions, the protein–solvent interactions are stronger in OPC.

## Discussion

Chignolin and CLN025 are among the most investigated fast-folding proteins concerning force field differences and testing various setups in computer simulations.^34,35,38,94,95^ Due to their small size and distinct native conformation, they present fast-folding kinetics and hence are ideal candidates for examining protein folding. Characterizing the folding dynamics of Chignolin/CLN025 can facilitate the understanding of larger, biologically relevant systems and can consequently inform the design and development of new biotherapeutics. We present a thorough atomistic characterization of the kinetically independent states and their transitions considering different force fields and water models (ff14SB/TIP3P and ff19SB/OPC).

As previously addressed by Kührová et al., Chignolin does not only adopt a natively folded state and unfolded conformations, but also forms a misfolded state, which is characterized by parallel shifted β-strands.^38^ In contrast to prior studies,^96^ we find similar results also for CLN025, however, an additional misfolded structure could be characterized. This other misfold is caused by steric hindrances of the terminal tyrosines, which explains its absence in Chignolin. The asymmetric character of the first misfolded state, present in Chignolin and CLN025, has already been outlined before.^38^ The additional misfolded state forms a more complex network with varying hydrogen bond partners (Y1–T8/W9/Y10 and D3–E5/T6/G7/T8). Although this state is missing stabilizing backbone interactions to form a secondary structure motif, it still is a preserved conformation with striking side chain hydrogen bonds and aromatic interactions (π-stacking Y2–Y10 and T-stacking W9–Y10). We sample the two different misfolded conformations in both simulations of CLN025, independent of the force field/water model combination (Figure S2). However, the separation was only possible in the ff14SB/TIP3P sampling due to the higher stability of folded states in this run.

Previous studies have already highlighted the difference in stability of the two variants, which is also apparent in this study.^38,39,96^ Due to the terminal tyrosines, CLN025 forms additional interactions, stabilizing the folded conformation. This was also emphasized in NMR studies of the proteins, where Chignolin was folded 60% of the time and CLN025 around 90% (both at 300 K).^37,39^ It has been previously demonstrated that ff14SB/TIP3P generally overstabilizes secondary structures, especially helices.^35,45,97^ Yet, if we compare the probabilities presented here, we find similar results for the folded conformation probability in ff14SB/TIP3P and the NMR experiments. The folded state of Chignolin is slightly overstabilized (66 ± 13% folded and 26 ± 4% misfolded compared to 60% folded in the experiment), but that of CLN025 is very similar (63 ± 1% folded and 24 ± 1% misfolded compared to 90% folded in the experiment). If we have a look at the different setup (ff19SB/OPC) in the same context, we find distinct differences, independent of the investigated system (Chignolin, 16 ± 1% folded and 5 ± 1% misfolded; CLN025, 31 ± 12% folded and 1.2 ± 0.3% misfolded). This hints at a higher stabilization of the unfolded states, possibly caused by stronger interactions of the water model with the protein.

Furthermore, the GIST results highlight that the mentioned aromatic residues are able to compensate for the desolvation penalty by forming additional stronger interactions with water, stabilizing the folded states. The strong increase in GIST enthalpy, i.e., higher interaction energies for the OPC, favors protein–water interactions, which might explain the higher population of the unfolded state. Supposedly the increased multipole values (i.e., dipole and quadrupole) of the OPC water model compared to TIP3P could be responsible for stabilizing unfolded conformations.^49^ The importance of the water model in determining thermodynamics and kinetics of the protein folding is further emphasized in Figures S4 and S5, showing that simulations with Chignolin using ff14SB/OPC and ff19SB/TIP3P reveal similar trends, i.e., higher probability of the unfolded state when using the OPC water model. Also other authors have found that OPC, in comparison to TIP3P, in some instances favors extended structures of proteins.^97,98^ Nevertheless, further testing of different proteins with the respective force fields and water models is crucial to identifying the strengths and weaknesses of the individual approaches and setups. Especially since Chignolin and CLN025 both feature the same secondary structure, namely, the β-hairpin, it is challenging to extrapolate the conclusions from these proteins to larger/structurally different systems. Nevertheless, a larger solvent accessible surface area may potentially lead to higher solvation free energy, if the water model exhibits particularly strong interactions with the solute. Accordingly, the influence might decrease with compactness and size due to a lack of target area. On the other hand, an overstabilization of unfolded conformations could also hold advantages in exploring a broader conformational space and sampling structurally distinct states in a shorter simulation time. This is in particular beneficial for larger proteins, where the transition times of large conformational changes can be very long.^11,99^ Furthermore, it has been demonstrated that ff19SB/OPC, presumably due to this overstabilization, is favorable for intrinsically disordered proteins. While other force field/water model combinations mainly adopt overly compact conformations relative to the experiment, this setup presents a more reasonable structural ensemble.^98^

Overall, it is rather difficult to propose a general recipe for simulating protein folding processes since there are multiple factors involved in addition to the critical role of the force field and the water model. One of the obstacles in characterizing protein folding processes are the time scale at which the folding events occur.^34,100^ To sample a thermodynamically and kinetically meaningful ensemble, numerous transitions between the unfolded and the folded states are necessary, which consequently increases the required simulation time. To accelerate the folding process and to overcome the time scale limitations of classical molecular dynamics simulations, enhanced sampling techniques (like hyperdynamics, replica-exchange, or metadynamics) and simulations at higher temperatures can be considered.^101−106^ However, to reconstruct the thermodynamics and kinetics of these folding events from biased trajectories, reweighting schemes need to be applied.^107^ Thus, the simulation parameters need to be chosen in accordance with the proposed research question, including the careful selection of the water model and force field.

This choice also plays an important role in the estimation of properties that are relevant for experimental studies. We have recently shown that the choice of the water model in molecular simulations may have a dramatic effect on the thermodynamics of complex conformational transitions, such as the Coil–Globule transition, which shares similarities with protein folding.^108^ Such biased ensembles will influence the calculated ensemble properties. For example, in structure refinement protocols, force fields and their respective water models are often used to optimize the final conformations based on experimental measures.^109^ One highly used experimental technique to identify folding events is NMR via the nuclear Overhauser effect (NOE) distances. Here, the spatial proximity of atoms/residues indicates crucial interactions and thus the three-dimensional conformation. Hence, these results can be compared to the native contact analysis, which also states the proximity of atoms in comparison to a reference structure and therefore informs about the folding state. Additionally, the *J*-coupling values from NMR results, i.e., via the Karplus equation, provide information about the backbone dihedrals and thus the conformational arrangement.^110−112^ This is especially important for the available structures of CLN025, since there are variations in the backbone torsions between the NMR and the crystal structure.^39^ In addition to the structural information, amide H-D exchange experiments are available for Chignolin^37^ and CLN025,^39^ which identified hydrogens in the backbone that are not surface-exposed and thus might contribute to form stabilizing hydrogen bonds. Therefore, the results from the H-D exchange experiments are suitable for comparison to the secondary structure estimation (also with the dihedrals from the *J*-coupling) and interaction analyses. In general, there are various experimental techniques which go hand-in-hand with computational analyses and thus represent valuable factors for comparison and validation that guide and drive advances in the field.

## Conclusion

We present an exhaustive comparison of the force fields ff14SB and ff19SB with their recommended water models, TIP3P and OPC, respectively, and characterize folding kinetics and thermodynamics of both Chignolin and CLN025. We described previously unidentified states in detail, regarding their conformations and intramolecular interaction patterns. The complexity of the state model and the transitions between states exemplifies the general challenge of understanding protein folding, unfolding, and misfolding.

By outlining their folding pathway in atomistic detail, we were able to differentiate the two systems, Chignolin and CLN025, according to their thermodynamic and kinetic behavior in solution. Comparing the different setups, namely, ff14SB/TIP3P and ff19SB/OPC, we find a population shift from folded to unfolded conformations. An in-depth characterization of this discrepancy points out a stabilizing effect of the unfolded state by the OPC water model independent of the force field. Thus, the choice of force field and especially the water model have a significant impact on the resulting conformational ensemble and determine physically relevant properties. To identify the most influential factors and to gain more comprehensive knowledge, further examples still need to be investigated. However, compared to the compact size of Chignolin, larger systems require a more extensive effort for convergence and come with a higher statistical uncertainty. Our findings could also have profound implications for drug discovery, where conformational variations less dramatic than folding–unfolding can occur between *apo* and *holo* transitions. In these scenarios, significantly different results could be obtained when assessing the energetic accessibility of ligand-able conformations in proteins of therapeutic relevance, depending on the force field and water model combinations used. To conclude, this study informs about important considerations when requiring kinetically and thermodynamically reasonable states reaching experimental agreement.

## Data Availability

The structures used in this manuscript are publicly available, with the PDB codes 1UAO and 5AWL. The trajectories have been made available via Zenodo (10.5281/zenodo.10499332).

## Supporting Information Available

Principal component analysis (PCA) and time-lagged independent component analysis (tICA) in color, extended hydrogen bond analysis, cross solvation results (ff14SB/OPC and ff19SB/TIP3P) for Chignolin, transition times, population probabilities, and Markov state model quality checks (PDF)
