# Supplementary material for: The Role of Force Fields and Water Models in Protein Folding and Unfolding Dynamics
Source: J Chem Theory Comput. 2024 Feb 19;20(5):2321–33. doi: 10.1021/acs.jctc.3c01106 (PMC10938642; doi:10.1021/acs.jctc.3c01106)
Supplement: Supplementary file 1 — ct3c01106_si_001.pdf [file ct3c01106_si_001.pdf]

Supporting Information

for

The role of force fields and water models in protein folding and unfolding dynamics

Anna-Lena M. Fischer[1], Anna Tichy[1], Janik Kokot[1], Valentin J. Hoerschinger[1], Robert F. Wild[1], Jakob R. Riccabona[1], Johannes R. Loeffler[1], Franz Waibl[2], Patrick K. Quoika[3], Philipp Gschwandtner[4], Stefano Forli[5], Andrew B. Ward[5], Klaus R. Liedl[1], Martin Zacharias[3], Monica L. Fernández-Quintero[1*]

[1] Institute for General, Inorganic and Theoretical Chemistry, Center for Molecular Biosciences Innsbruck (CMBI), University of Innsbruck, A-6020 Innsbruck, Austria

[2] Department of Chemistry and Applied Biosciences, ETH Zürich, Vladimir-Prelog-Weg 2, 8093 Zürich, Switzerland

[3] Center for Protein Assemblies (CPA), Physics Department, Chair of Theoretical Biophysics, Technical University of Munich, D-80333 Munich, Germany

[4] Research Center HPC, University of Innsbruck, A-6020 Innsbruck, Austria

[5]Department of Integrative Structural and Computational Biology, Scripps Research Institute, La Jolla, CA 92037, USA

*Corresponding author: Monica Fernández-Quintero; monica.fernandez-quintero@uibk.ac.at

Free energy surfaces of Chignolin and CLN025: PCA and tICA

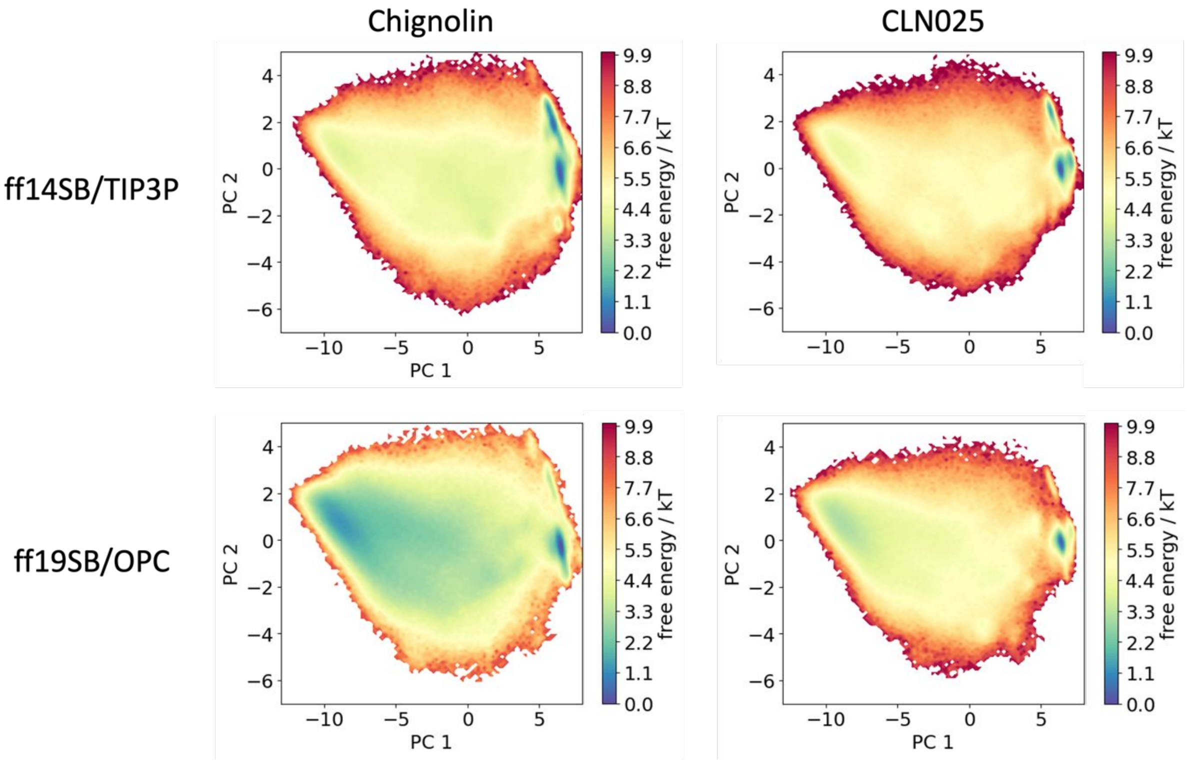

S1: Principal Component Analysis (PCA) of Chignolin and CLN025 of the two different force field/water model combinations (ff14SB/TIP3P and ff19SB/OPC) projected in the combined space. It is clearly visible, that the simulations cover the same area on the free energy surface.

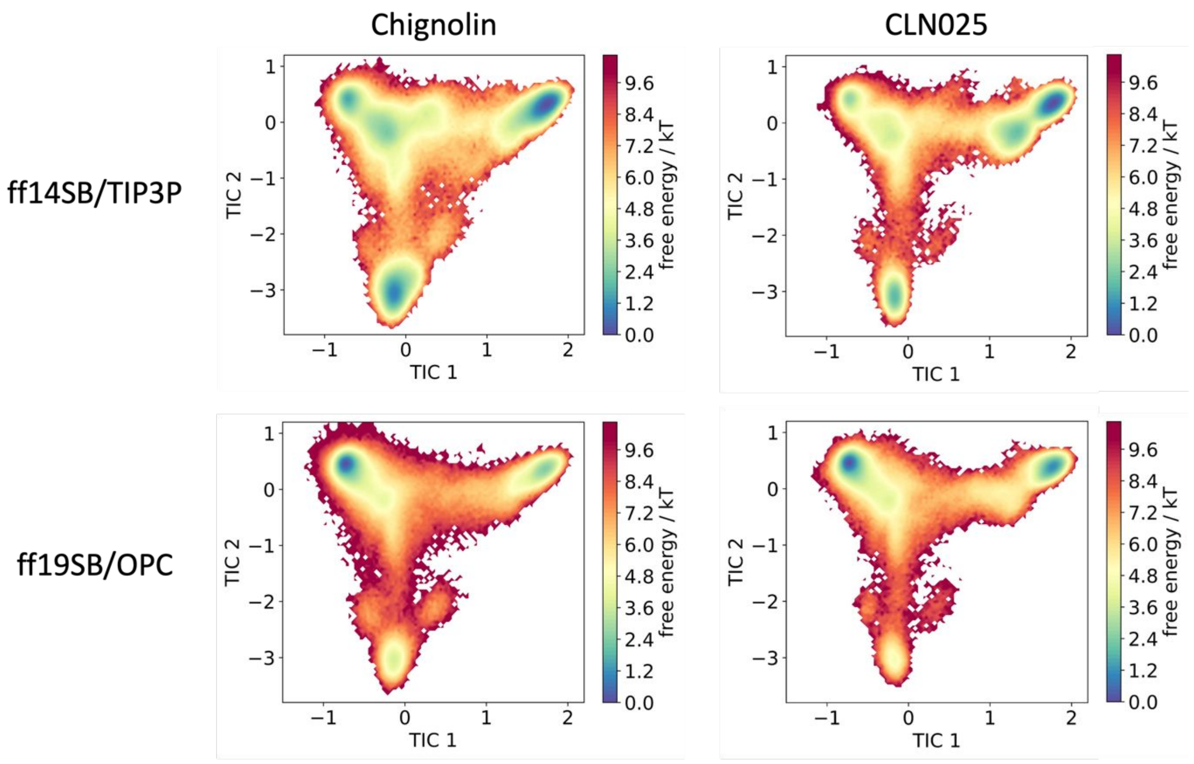

S2: Time-lagged Independent Component Analysis (tICA) of Chignolin and CLN025 of the two different force field/water model combinations (ff14SB/TIP3P and ff19SB/OPC) projected in the combined space. The different depth of the minima demonstrates the population of the corresponding states.

Extended hydrogen bond analysis

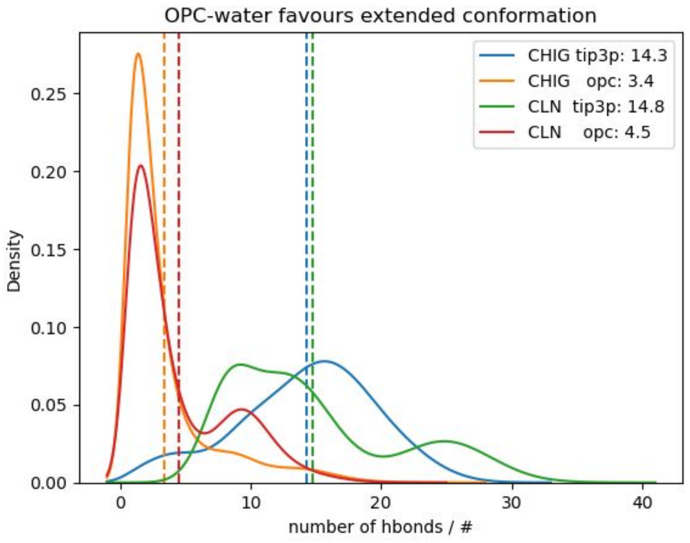

S3: Distribution of the total number of intramolecular hydrogen bonds during the simulation. Orange and red are the simulations with ff19SB/OPC, blue and green with ff14SB/TIP3P. The dotted lines represent the averages, which are also highlighted in numbers in the plot-legend.

Cross solvation simulations of Chignolin: ff14SB/OPC and ff19SB/TIP3P

We again find a significant shift in probabilities of folded and unfolded state depending on the water model. While for the simulation with TIP3P the folded state is the highest populated (S5) for the one with OPC the unfolded state is favoured (S4).

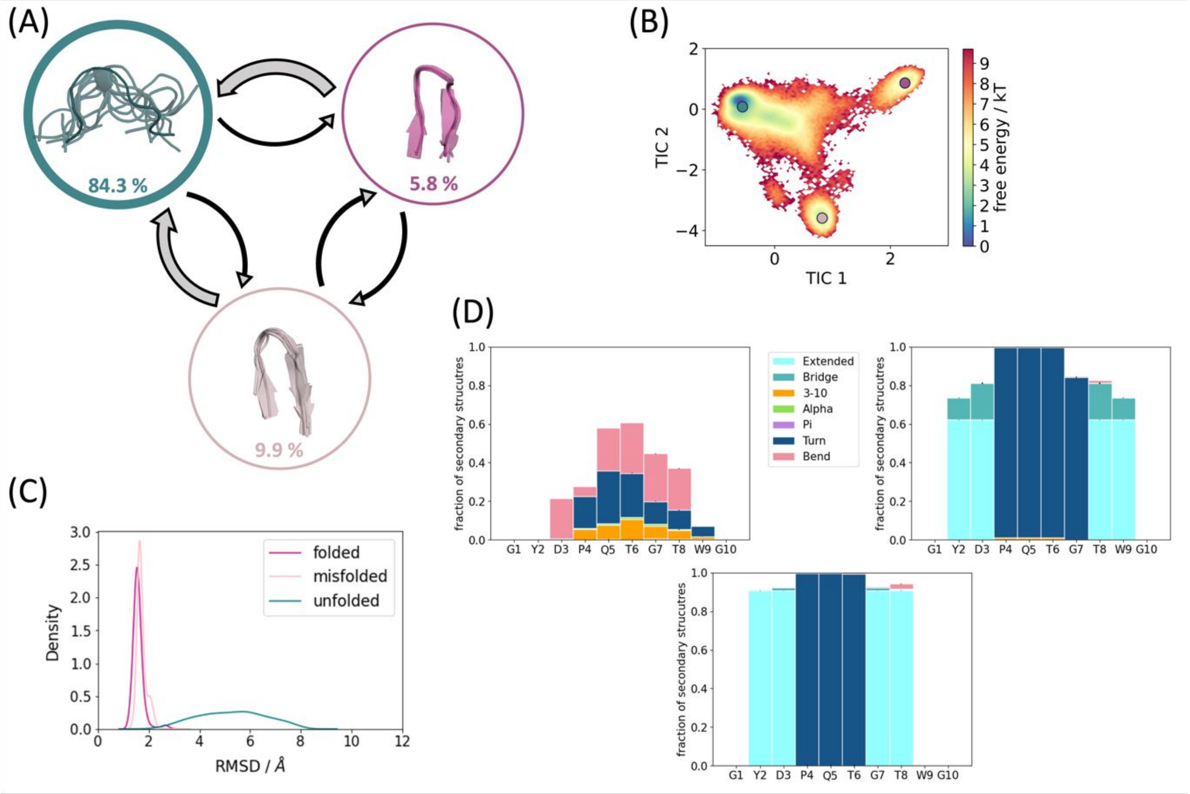

S4: Analysis of cross solvation simulations ff14SB/OPC of Chignolin. (A) shows the population of the macrostates with their transition times. Green is the unfolded state, purple the natively folded and light pink are the misfolded states. Errors of the probabilities as well as the transition times and the MSM validations are shown in Table 5 and 7 and Figure S7. (B) depicts the free energy landscape (tICA) with the macrostate position indicated via dots in the respective color. (B) represents the RMSDs of the states. In (D) is the secondary structures for each state displayed determined according to DSSP.

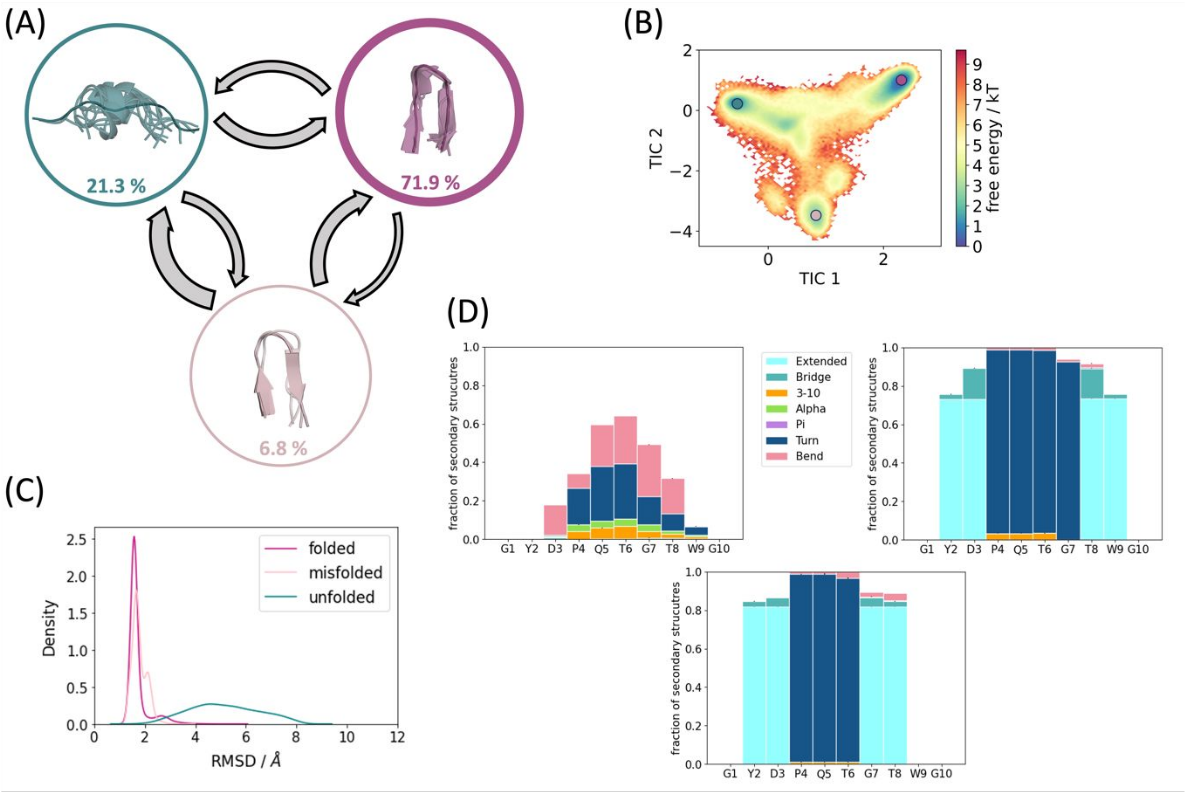

S5: Analysis of cross solvation simulations ff19SB/TIP3P of Chignolin. (A) shows the population of the macrostates with their transition times. Green is the unfolded state, purple the natively folded and light pink are the misfolded states. Errors of the probabilities as well as the transition times and the MSM validations are shown in Table 6 and 7 and Figure S7. (B) depicts the free energy landscape (tICA) with the macrostate position indicated via dots in the respective color. (B) represents the RMSDs of the states. In (D) is the secondary structures for each state displayed determined according to DSSP.

Transition times, populations and validations of Markov state models

Table 1: Transition times in between macrostates of Chignolin with ff14SB/TIP3P. The row is always transitioning to the column.

transition times	natively folded	misfolded	unfolded
natively folded	-	1.0 ± 0.02 µs	600 ± 10 ns
misfolded	400 ± 10 ns	-	600 ± 20 ns
unfolded	100 ± 10 ns	600 ± 10 ns	-

Table 2: Transition times in between macrostates of Chignolin with ff19SB/OPC. The row is always transitioning to the column.

transition times	natively folded	misfolded	unfolded
natively folded	-	5.5 ± 0.2 µs	600 ± 20 ns
misfolded	2.2 ± 0.1 µs	-	300 ± 10 ns
unfolded	2.7 ± 0.1 µs	5.6 ± 0.2 µs	-

Table 3: Transition times in between macrostates of CLN025 with ff14SB/TIP3P. The row is always transitioning to the column.

transition times	natively folded	misfolded (light pink)	misfolded (pink)	unfolded
natively folded	-	7.1 ± 0.4 µs	4.1 ± 0.2 µs	2.2 ± 0.1 µs
misfolded (light pink)	1.1 ± 0.1 µs	-	3.7 ± 0.2 µs	700 ± 50 ns
misfolded (pink)	600 ± 30 ns	6.3 ± 0.4 µs	-	1.3 ± 0.1 µs
unfolded	600 ± 30 ns	4.9 ± 0.2 µs	3.2 ± 0.2 µs	-

Table 4: Transition times in between macrostates of CLN025 with ff19SB/OPC. The row is always transitioning to the column.

transition times	natively folded	misfolded	unfolded
natively folded	-	16.9 ± 1.3 µs	2.5 ± 0.2 µs
misfolded	4.1 ± 0.2 µs	-	200 ± 40 ns
unfolded	4.3 ± 0.2 µs	14.4 ± 1.3 µs	-

Table 5: Transition times in between macrostates of Chignolin with ff14SB/OPC. The row is always transitioning to the column.

transition times	natively folded	misfolded	unfolded
natively folded	-	3.6 ± 0.2 µs	200 ± 20 ns
misfolded	5.3 ± 0.5 µs	-	500 ± 30 ns
unfolded	4.8 ± 0.5 µs	3.3 ± 0.2 µs	-

Table 6: Transition times in between macrostates of Chignolin with ff19SB/TIP3P. The row is always transitioning to the column.

transition times	natively folded	misfolded	unfolded
natively folded	-	800 ± 30 ns	300 ± 70 ns
misfolded	200 ± 10 ns	-	100 ± 10 ns
unfolded	200 ± 10 ns	300 ± 10 ns	-

Table 7: State probabilities of the macrostates with their error estimates according to block averaging.

state probability	natively folded	misfolded (light pink)	misfolded (pink)	unfolded
Chignolin ff14SB/TIP3P	65.6 ± 13.0 %	26.2 ± 4.2 %	-	8.2 ± 11.4 %
Chignolin ff19SB/OPC	15.9 ± 1.0 %	4.7 ± 1.0 %	-	79.38 ± 0.02 %
CLN025 ff14SB/TIP3P	63.0 ± 1.3 %	11.1 ± 0.4 %	12.6 ± 0.8 %	13.3 ± 0.8 %
CLN025 ff19SB/OPC	31.3 ± 11.6 %	1.2 ± 0.3 %	-	67.5 ± 11.3 %
Chignolin ff14/OPC	5.8± 1.0 %	9.9± 1.7%	-	84.3 ± 2.0 %
Chignolin ff19/TIP3P	71.9 ± 13.5 %	6.8 ± 4.6 %	-	21.3 ± 11.8 %

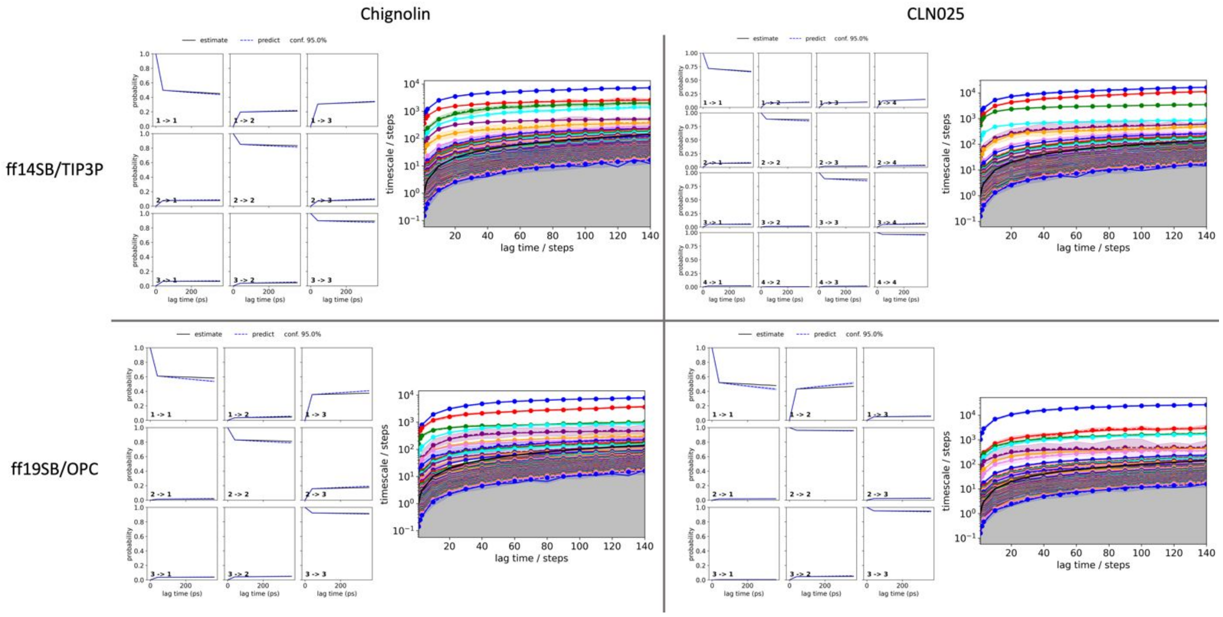

S6: Markov state quality checks for all MSMs. On the left is the Chapman-Kolmogorov Test to check our model for Markovianity, on the right are the implied timescales which indicate the number of metastable states according to gaps in between.

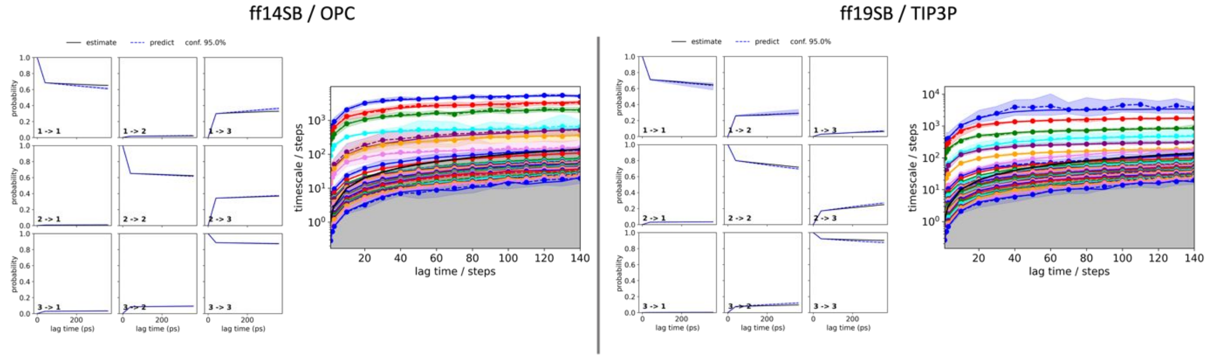

S7: Markov state quality checks for cross solvation MSMs (ff14SB/OPC and ff19SB/TIP3P). On the left is the Chapman-Kolmogorov Test to check our model for Markovianity, on the right are the implied timescales which indicate the number of metastable states according to gaps in between.
